# A Cost-Effectiveness Analysis of Gemcitabine plus Cisplatin Versus Gemcitabine Alone for Treatment of Advanced Biliary Tract Cancer in Japan

**DOI:** 10.1007/s12029-016-9885-6

**Published:** 2016-10-26

**Authors:** Ikuto Tsukiyama, Masayuki Ejiri, Yoshihiro Yamamoto, Haruhisa Nakao, Masashi Yoneda, Katsuhiko Matsuura, Ichiro Arakawa, Hiroko Saito, Tadao Inoue

**Affiliations:** 1 Department of Pharmacy, Aichi Medical University Hospital, 1-1 Yazago Karimata, Nagakute-shi, Aichi 480-1195 Japan; 2 Faculty of Pharmacy, Meijo University, Nagoya, Japan; 3 Division of Gastroenterology, Department of Internal Medicine, Aichi Medical University, School of Medicine, Nagakute-shi, Japan; 4 Faculty of Pharmaceutical Science, Teikyo Heisei University, Tokyo, Japan; 5 School of Pharmaceutical Sciences, Ohu University, Koriyama, Hukushima Japan

**Keywords:** Gemcitabine, Cisplatin, Combination therapy, Cost-effectiveness, Advanced biliary tract cancer, Japan

## Abstract

**Objectives:**

This study assessed the cost-effectiveness of combination treatment with gemcitabine and cisplatin compared to treatment with gemcitabine alone for advanced biliary tract cancer (BTC) in Japan.

**Methods:**

A monthly transmitted Markov model of three states was constructed based on the Japan BT-22 trial. Transition probabilities among the health states were derived from a trial conducted in Japan and converted to appropriate parameters for our model. The associated cost components, obtained from a receipt-based survey undertaken at the Aichi Medical University Hospital, were those related to inpatient care, outpatient care, and treatment for BTC. Costs for palliative care and treatment of adverse events were obtained from the National Health Insurance price list. We estimated cost-effectiveness per quality-adjusted life year (QALY) at a time horizon of 36 months. An annual discount of 3 % for both cost and outcome was considered.

**Results:**

The base case outcomes indicated that combination therapy was less cost-effective than monotherapy when the incremental cost-effectiveness ratio (ICER) was approximately 14 million yen per QALY gained. The deterministic sensitivity analysis of the ICER revealed that the ICER of the base case was robust. A probabilistic analysis conducted with 10,000-time Monte Carlo simulations demonstrated efficacy at the willingness to pay threshold of 6 million yen per QALY gained for approximately 33 % of the population.

**Conclusion:**

In Japan, combination therapy is less cost-effective than monotherapy for treating advanced BTC, regardless of the statistical significance of the two therapies. Useful information on the cost-effectiveness of chemotherapy is much needed for the treatment of advanced BTC in Japan.

## Introduction

The incidence rate of biliary tract cancer (BTC) in the Japanese population exceeds that in the US, European, and East Asian populations. In 2015, approximately 27,000 cases of BTC, the eighth most frequent cause of mortality from cancer, occurred in Japan [1]. Well-known risk factors for BTC include biliary tract diseases, such as cholelithiasis, and inflammation of the gallbladder and biliary tract. Recently, there has been much concern about the high incidence of BTC in workers at printing presses because of their exposure to 1,2-dichloropropane, which has been implicated as a possible cause of BTC.

Gemcitabine and fluoropyrimidine drugs are commonly used as systemic chemotherapy for advanced BTC. In addition, a meta-analysis of 112 clinical trials concluded that combination treatment with gemcitabine and a platinum agent was effective for the treatment of BTC [2]. Furthermore, two randomized controlled trials (RCTs) (UK ABC-01 [3]) demonstrated that gemcitabine plus cisplatin (GC combination therapy) was an effective treatment for BTC. A phase III trial (UK ABC-02 (4)) was carried out in the UK to investigate the clinical efficacy and safety of GC combination therapy (1000 mg/m^2^ of gemcitabine + 25 mg/m^2^ of cisplatin on days 1 and 8, repeated every 3 weeks) versus gemcitabine alone (G monotherapy) (1000 mg/m^2^ of gemcitabine on days 1, 8, and 15, repeated every 4 weeks) on the primary endpoints of overall survival (OS) and progression-free survival. In the ABC-02 study [4], the treatments resulted in median OS times of 11.7 and 8.1 months, respectively, and progression-free survival times of 8 and 5 months, respectively. Using the same protocol as the ABC-02 study [4], an RCT called the BT-22 trial was conducted in Japan [5]. The results indicated that the median OS times for GC combination therapy and G monotherapy were not significantly different (11.2 and 7.7 months (*p* = 0.139), respectively), whereas the progression-free survival times were not significantly different (5.8 and 3.7 months (*p* = 0.077), respectively). These findings agreed with the results of the ABC-02 study [4]. The updated guidelines for Japan, therefore, recommend GC combination therapy as first-line treatment for advanced BTC.

Roth et al. [6] evaluated the cost utility of GC combination therapy in the USA using the results of the ABC-02 study [4] and concluded that GC combination therapy was more cost-effective than G monotherapy as per the accepted standards of willingness to pay (WTP) in the USA (50,000 US dollars per quality-adjusted life year (QALY) gained).

Using the results of the BT-22 trial [5], this study assessed the cost-effectiveness of GC combination therapy compared to that of G monotherapy for the treatment of BTC from the perspective of healthcare payers in Japan.

## Methods

### Model Building

**Fig. 1:**
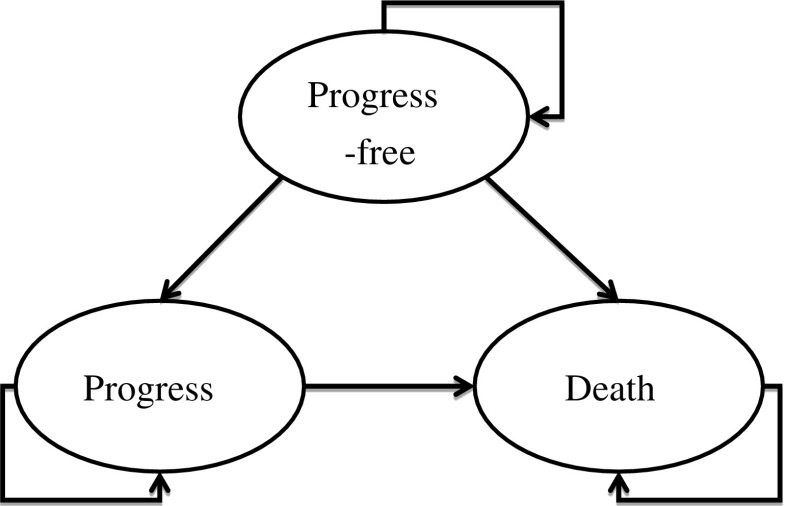
A simple and three-state Markov model on the cost-effectiveness of the GC combination therapy versus G monotherapy

A Markov model comprising three simple monthly transmitted states (no progress, progress, and death) was implemented using the results of the BT-22 trial [5] (Fig. 1). The transition probabilities among these health states were derived from data from the BT-22 trial [5] and converted to appropriate parameters for our model (see Eq. 1 and Table 1).

1 $$ p=1-{e}^{-rt}, $$

**Table 1:** Parameters incorporated in the model

Parameter	Distribution	Mean	SD	*N*	Ref.
Adverse events in G only	Beta	0.688	0.07	206	[1]
Adverse events in combination	Beta	0.707	0.071	206	[1]
Progress rate in combination	Beta	0.1126	0.0494	41	[2]
Progress rate in G only	Beta	0.1708	0.0581	42	[2]
Mortality rate in combination	Beta	0.0600	0.0162	41	[2]
Mortality rate in G only	Beta	0.0861	0.0433	42	[2]
Utility in no progress	Gamma	0.690	0.120	-	[7]
Utility in progress	Gamma	0.710	0.130	-	[7]
Utility in adverse events	Gamma	0.678	0.120	-	[7]
Monthly outpatient cost	Gamma	35,148	22,402	-	Receipt survey
Monthly inpatient cost	Gamma	212,990	104,633	-	
Parameter	Distribution	Minimum	Most likely	Maximum	
Monthly palliative cost	Triangular	1,330,020	1,501,200	1,625,580	NHIP 2012 rev.
Monthly cost on G-CSF agent	Triangular	24,852	27,613	30,374	
Monthly drug cost in G only	Triangular	39,552	43,947	48,342	
Monthly drug cost in comb	Triangular	40,117	44,574	49,031	

where *p* was the probability, *r* was the hazard ratio (HR), and *t* was the time (months).

To calibrate and validate this model, the HR for GC combination therapy versus G monotherapy for OS was externally generated. This HR was then compared to that reported in the BT-22 trial [5] and the resulting survival curve was drawn [2].

### Cost Variables

**Fig. 2:**
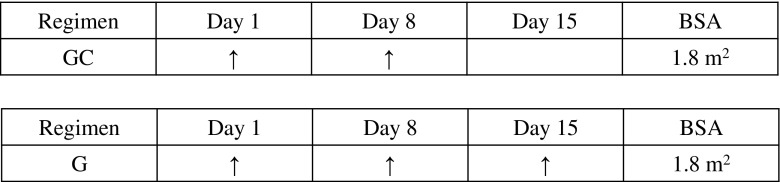
Regimen for treatment of advanced BTC. *BSA* body surface area, *GC* gemcitabine plus cisplatin, *G* gemcitabine

BTC-associated cost components were obtained from a receipt-based survey of ten subjects treated at the Aichi Medical University Hospital. The components were set as inpatient costs, outpatient costs, medication costs for BTC, and treatment costs for palliative care (Table 1). An ethics approval for the survey was obtained in advance from the Medical Institute (Approval no.: 13-034). In addition, when considering the cost of palliative care in Japan, dietary care is commonly included. According to the National Health Insurance Price (NHIP) list, the daily unit cost is 49,260 yen for palliative care and 780 yen for meals. We, therefore, estimated a total monthly cost of 1,501,200 yen by multiplying the summed daily unit cost by 30 days. The cost of the chemotherapy drugs (gemcitabine and cisplatin) and of treatment for adverse events (namely, granulocyte-colony stimulating factor (G-CSF) therapy for hematotoxicity) was calculated based on the NHIP list (2012 revision) cost for each drug (Fig. 2).

### Health Outcomes

QALY was defined as the primary health outcome. The utility weights for the health states were devised using the EuroQol Five Dimension (EQ-5D) questionnaire (Table 1) and incorporated into the model. We also referred to the Cost-Effectiveness Analysis (CEA) Registry [7], established by the Center for the Evaluation of Value and Risk in Health (CEVR) at the Tufts Medical Center, because the appropriate utility weights for BTC were not available in the Japanese context.

### Cost-Effectiveness Assessment

The cost-effectiveness of GC combination therapy compared to that of G monotherapy was assessed using the incremental cost-effectiveness ratio (ICER) at a time horizon of 36 months. An annual discount of 3 % was considered for both cost and outcome. The health technology was considered cost-effective from the perspective of healthcare payers (excluding patient-time cost), if the ICER was less than the WTP threshold of 6 million yen per QALY (converted to 500,000 yen per quality-adjusted life month (QALM)) gained.

### Sensitivity Analysis

To include uncertainty in the simulated cost-effectiveness analysis, we conducted deterministic and probabilistic uncertainty analyses using a tornado diagram and Monte Carlo simulation, respectively. The tornado diagram revealed the parameters that influenced the base case and also demonstrated its robustness. In addition, a Bayesian approach was applied to the probabilistic analysis of the Markov model, in which we used 10,000-time Monte Carlo simulations to choose an efficient strategy for the aforementioned WTP threshold of 6 million yen per QALY gained. The chosen distribution for each of the parameters is indicated in Table 1. In principal, continuous variables, such as the interval scale, were assumed to follow a gamma distribution. Drug cost was assumed to follow a triangular distribution, which is useful as an approximate model if there are no appropriate values available for analysis. A minimum value *a* (most likely value minus 10 %), the most likely value *m*, and a maximum value *b* (most likely value plus 10 %) were specified. The triangular variable *x* was assumed to have the probability density function seen in Eq. 2:

2 $$ \left.\begin{array}{cc}\hfill f(x)=\frac{2\left(x-a\right)}{\left(b-a\right)\left(m-a\right)}\hfill & \hfill \mathrm{if}\ a<x<m\hfill \\ {}\hfill f(x)=\frac{2\left(b-x\right)}{\left(b-a\right)\left(m-b\right)}\hfill & \hfill \mathrm{if}\ m\le x<b\hfill \end{array}\right] $$

Variables with binomial distributions were assumed to follow a beta distribution. Beta [*r, n*] such that

3 $$ \mathrm{mean}=\frac{r}{n}.\kern0.5em SD=\sqrt{\frac{r\left(n-r\right)}{n^2\left(n+1\right)},} $$

where *r* is the event from *n* samples, and SD denotes standard deviation.

## Results

### Model Calibration and External Validation

**Fig. 3:**
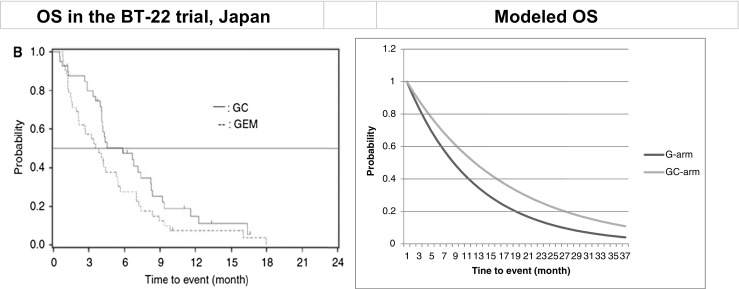
Model calibration and external validation. Real estimated HR 0.69 (0.41–1.13), modeled HR 0.688. Real reported survival time 11.2 versus 7.7 months (GC versus G), modeled time 11.2 versus 7.7 months

The survival curve is illustrated in Fig. 3. In terms of OS, the model demonstrated no statistical significance for the HR of 0.688. In comparison, a real estimated HR of 0.69 (95 % confidence interval 0.41–1.13, not significant) was retrieved from the clinical trial [5].

### Cost-Effectiveness for the Base Case

The incremental cost was approximately 3 million yen, even though the incremental effectiveness showed a gain of 273 QALMs. Using the relationship illustrated in Eq. 4, we calculated the ICER to be approximately 1.1 million yen per QALM gained (converted to 13 million yen per QALY gained), indicating G monotherapy was more cost-effective. In contrast, the base case outcome indicated that GC combination therapy was more effective than G monotherapy (Table 2).

**Table 2:** Base case for cost per QALYs gained

	Gemcitabine plus cisplatin	Gemcitabine only	Incremental
Cost (yen)	15,446,575	12,328,228	3,118,347
Eff. (QALMs)	10.04	7.61	2.73

Incremental cost-effectiveness ratio (ICER) = 3 yen, 118,347/2.73 QALMs = 13,707,020 yen/QALY gained >6,000,000/QALYs gained (not cost-effective)

*QALY* (*M*) quality-adjusted life year (month)

4 $$ \begin{array}{c}\hfill 3,118,347\ \mathrm{yen}\ \mathrm{per}\ 2.73\ \mathrm{QALMs}\kern0.75em =\kern0.75em 3,118,347\ \mathrm{yen}\ \mathrm{per}\ 2.73\ \mathrm{QALMs}/12\kern0.5em \mathrm{months}\ \left(\mathrm{converted}\ \mathrm{from}\ \mathrm{months}\ to\ \mathrm{years}\right)=3,118,347\ \mathrm{yen}\ \mathrm{per}\ 0.2275\ \mathrm{QALY}\mathrm{s}\hfill \\ {}\hfill =13,707,020\ \mathrm{yen}\ \mathrm{per}\ \mathrm{QALY}\ \mathrm{gained}\hfill \end{array} $$

### Deterministic and Probabilistic Sensitivity Analyses

**Fig. 4:**
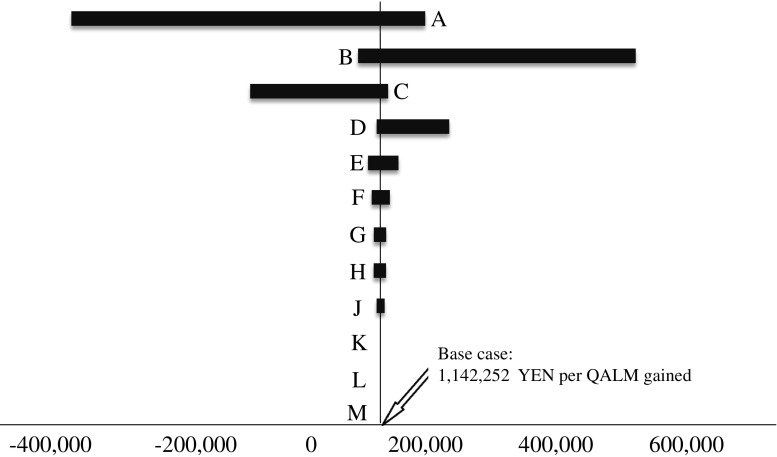
Tornado diagram for deterministic sensitivity analysis. *A* Probability of death in the GC group (0–0.083). *B* Probability of death in the G group (5–0.129). *C* Probability of progression-free survival in the GC group (0.045–0.119). *D* Probability of progression-free survival in the G group (0.045–0.119). *E* Utility of progression-free survival (0.445–0.965). *F* Utility of an adverse event heath state (0.443–0.913). *G* Monthly discount (8.0 × 10^−4^–0.0043). *H* Cost of palliative care (1,330,020–1,625,580 yen). *J* Utility of pre-progress (0.445–0.925). *K* Inpatient cost (191,691–234,289 yen). *L* Probability of any adverse events in the GC group (0.55–0.826). *M* Probability of any adverse events in the G group (0.58–0.846)

The tornado diagram depicting the deterministic sensitivity analysis of the ICER revealed that the death rate resulting from GC combination therapy influenced the base case. However, the robustness of the base case was confirmed (Fig. 4).

**Fig. 5:**
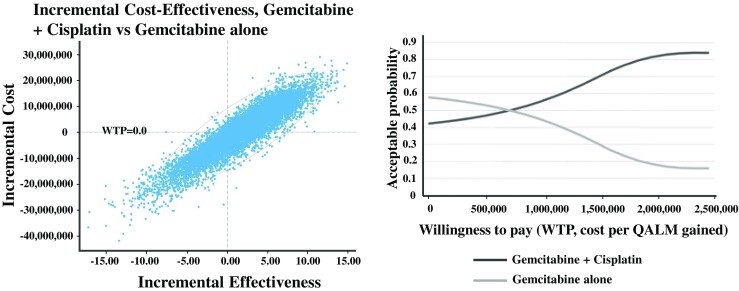
Probabilistic analysis with 10,000-time Monte Carlo simulations

The probabilistic analysis resulting from the 10,000-time Monte Carlo simulations demonstrated efficacy at a WTP threshold of less than 6 million yen per QALY gained by approximately 33 % of the population (Fig. 5).

## Discussion

We assessed the cost-effectiveness of GC combination therapy compared to that of G monotherapy for BTC from the perspective of healthcare payers in Japan and concluded that G monotherapy was more cost-effective. Based on the results of the ABC-02 trial [4], Roth et al. [6] concluded that GC combination therapy was more cost-effective than G monotherapy as per the accepted standards of WTP in the USA (50,000 US dollars per QALY gained). The discrepancy between the results of our study (for Japan) and those of Roth’s study (for the USA) existed because Roth’s analysis included patient-time costs, whereas ours did not. Roth considered the perspective of the society, whereas our study considered only the perspective of Japanese healthcare payers.

The constructed Markov model was validated by confirmation that the modeled survival curve and HR matched the real estimated value. The study design of the BT-22 trial [5] was the same as that of the ABC-02 trial [5], which used estimates of variables for the Markov state transmission model. Although the OS for GC combination therapy and G monotherapy in the BT-22 trial [5] was 11.2 and 7.7 months, respectively, and that in the ABC-02 trial [4] was 11.7 and 8.5 months, respectively, the BT-22 trial was chosen for estimating the probabilities of the Markov model because the BT-22 trial conducted an economic evaluation in the Japanese context, that is, it used data representative of Japan.

Even though GC combination therapy was less cost-effective than G monotherapy in our analysis, the reliability of extending patient survival time by approximately 3 months by adding cisplatin to gemcitabine (compared to G monotherapy) was significantly demonstrated in both the ABC-02 [4] and the BT-22 [5] trials. The expanded survival time may have increased medical costs by requiring additional treatment for increased side effects associated with adding cisplatin to gemcitabine and by the requirement for longer-term palliative care. In fact, the sensitivity analysis using the tornado diagram revealed that the cost of palliative care influenced the cost-effectiveness of GC combination therapy in our study. Therefore, GC combination therapy was less cost-effective than G monotherapy from the perspective of the healthcare payers because the extension of survival time led to an increase in medical costs.

The OS after GC combination therapy and G monotherapy in the ABC-02 trial was 8.2 and 6.5 months, respectively, both times exceeding the OS of 11.2 and 7.7 months from the BT-22 trial. Because long-term palliative care in Japan would cost more than that in the USA, the results of the cost-effectiveness analysis in the Japanese context would differ from that in the USA-based study. The guidelines for the treatment of advanced BTC in Japan recommend GC combination therapy as first-line therapy. The fact that this therapy is more effective and costlier should be specifically conveyed to patients before administering the combined medication. Our study suggested that G monotherapy is a better treatment strategy for advanced BTC than the guideline-recommended GC combination therapy by evaluation of the cost to healthcare payers in Japan.

Our study had several limitations with regard to the interpretation of the results. First, the value for QALYs was estimated by incorporating the utility weights provided by EQ-5D and derived from the CEA Registry established by the CEVR at the Tufts Medical Center [7]. The value was, therefore, not specific to the Japanese population. Where appropriate utility weights for Japanese patients with BTC were unavailable, data regarding hepatocellular carcinoma from the study by Roth et al. [6] were used. Nonetheless, we ensured the applicability of the utility weights by varying the range between the lower and upper values of the 95 % confidence interval while conducting the deterministic sensitivity analysis. Second, data on the cost variables were obtained by referring to the medical receipts of only a few patients from the Aichi Medical University. Additional data in this regard would help refine the results of future studies in this area. Third, not all patients in the real world may benefit from the cost-effectiveness indicated by the simulation results. The results of the 10,000-time Monte Carlo simulations indicated that approximately 40 % of cases may benefit from more cost-effective treatment.

## Conclusion

GC combination therapy was predicted to be less cost-effective than G monotherapy for treating advanced BTC in Japan. Practitioners should consider the possible cost benefits of GC combination therapy before patients suffering from advanced BTC undergo chemotherapy.
